# Development and Validation of an Individualized Nomogram for Predicting Survival in Patients with Esophageal Carcinoma after Resection

**DOI:** 10.7150/jca.40767

**Published:** 2020-04-06

**Authors:** Feng Du, Zhiwei Sun, Jun Jia, Ying Yang, Jing Yu, Youwu Shi, Bo Jia, Jiuda Zhao, Xiaodong Zhang

**Affiliations:** 1 Key Laboratory of Carcinogenesis and Translational Research (Ministry of Education/Beijing), The VIPII Gastrointestinal Cancer Division of Medical Department, Peking University Cancer Hospital and Institute, Beijing, China; 2 Key Laboratory of Carcinogenesis and Translational Research (Ministry of Education /Beijing), Department of Thoracic Medical oncology, Peking University Cancer Hospital and Institute, Beijing, China; 3 Affiliated Hospital of Qinghai University, High Altitude Medical Research Center, Xining, China

**Keywords:** Esophageal Neoplasms, Nomogram, Prognosis

## Abstract

An accurate estimation of prognosis of the esophageal carcinoma patients after surgery is urgently needed. Clinical nomogram has been developed to quantify risk by incorporating prognostic factors for individual patient. Based on the Surveillance, Epidemiology, and End Results (SEER) database from 2004 to 2013, a total of 4566 patients were selected. Of those, 3198 patients were assigned to training set to construct the nomogram, which incorporated age, gender, histology, grade, T stage, N stage, nodes examined, radiation and chemotherapy. The calibration curve for probability of survival showed good agreement between prediction by nomogram and actual observation. The C-index of the nomogram was 0.71(95%CI 0.70-0.72), which was statistically higher than the TNM staging system. The results were then validated using bootstrap resampling and a validation set of 1368 patients in the SEER database. Besides, in the esophageal squamous cell carcinoma and esophageal adenocarcinoma subgroups, the nomogram discrimination was superior to the TNM staging system. It is likely that these results would play a supplementary role in the current staging system and help to identify the high risk population after surgery.

## Introduction

Esophageal cancer represents a heterogenous entity that is associated with high morbidity and mortality. It is the eighth most common cancer worldwide, with roughly 450,000 new cases reported per year 1. Also, it ranks as the sixth most common cause of cancer-related death worldwide, with more than 80% of patients eventually succumbing to this disease 2.

Surgery is the primary treatment for patients diagnosed with resectable esophageal carcinoma. Nonetheless, the 5-year survival rate remains relatively modest at less than 40%.^3^, ^4^ It is of great importance to define the patient populations with higher risk to relapse or metastasis, who may receive more benefit from post-operative therapy.

An accurate estimation of survival rates of the esophageal carcinoma patients after surgery is therefore needed. The tumor node metastasis (TNM) classification is the most widely used staging system. However, several important prognostic factors, such as age and number of examined lymph nodes, are not included in TNM system 5. In addition, increasing evidences showed the unsatisfactory discriminative ability of TNM system in prognostic prediction 6-8.

Clinical nomogram has been developed with intuitive graphs to quantify risk by incorporating all known prognostic factors for individual patient 9. Nomogram has been widely used in different cancer types, and shown to be more accurate than the TNM staging systems for predicting prognosis 10-12.

The present study was designed to develop a prognostic nomogram for patients with non-metastatic esophageal carcinoma (nMEC) who underwent surgery based on National Cancer Institute^,^s Surveillance, Epidemiology, and End Results (SEER) database, to determine whether this model provided more-accurate prediction of patient survival when compared with TNM system. In addition, we assessed the performance of this model in esophageal adenocarcinoma (EAC) and esophageal squamous cell carcinoma (ESCC) population, respectively.

## Materials and Methods

### Data

The Surveillance, Epidemiology, and End Results (SEER) database is a population-based cancer registry that included a sample (about 27.8%) of the national population. We used the data based on the recent SEER 18 registries research database from 2004 to 2013.

We collected information on patient characteristics (age, gender and race), primary tumor features (location, histology, grade, T stage, N stage and nodes examined), treatment approaches (radiation and chemotherapy) and clinical outcomes (cancer specific survival and overall survival).

### Inclusion Criteria

We selected patients from the SEER database following International Classification Disease for Oncology, 3rd Edition (ICD-O3) topography codes for anatomic location in the esophagus: proximal esophagus (15.0 and 15.3), midthoracic esophagus (15.1 and 15.4), distal esophagus (15.2 and 15.5), overlapping lesions (15.8) and esophageal lesions, not otherwise specified (15.9).

We only included patients over the age of 18 who are histologically confirmed positive as esophageal malignant tumor. The following histologic subtypes were included (1) adenocarcinoma (8050-8052, 8123, 8140-8147, 8210-8211, 8255, 8260-8263, 8310, 8480-8481, 8490, 8550, 8570-8575) (2) squamous cell carcinomas (8032, 8070-8077, 8083, 8094).

### Exclusion Criteria

**Figure 1:**
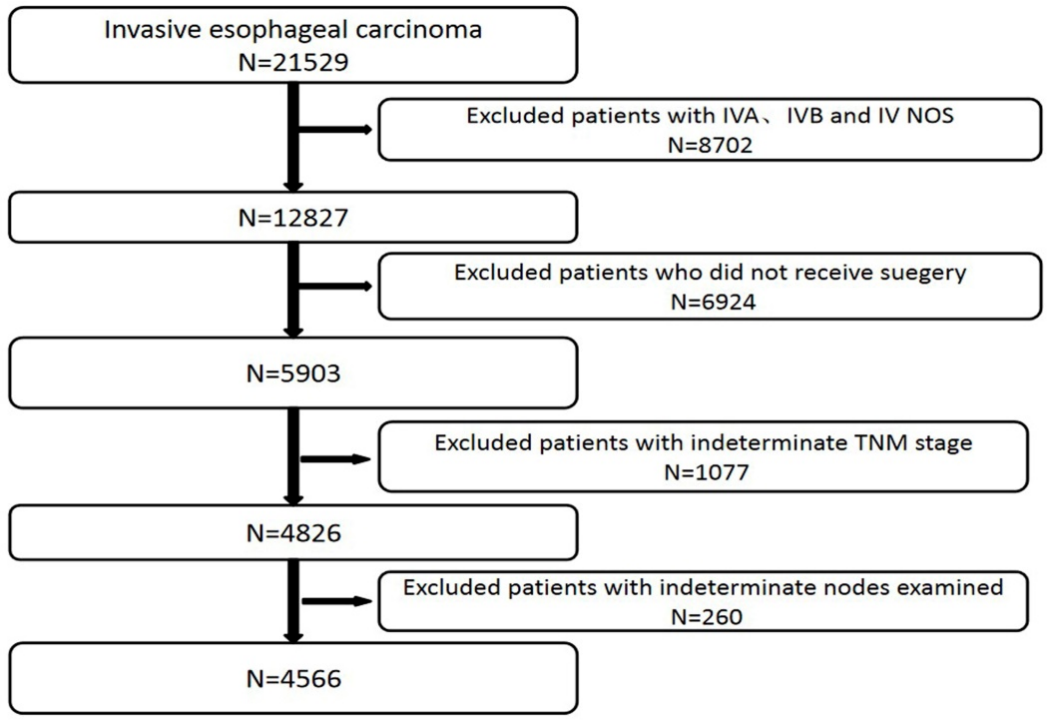
Patients included in and excluded from study.

We excluded patients with IVA, IVB and IV NOS stage (n=8702), patients who did not receive surgery (n=6924), patients with indeterminate TNM stage (n=1077) and patients with indeterminate nodes examined (n=260) (Figure 1). We also excluded those cases with any missing or unknown information in terms of the prognostic factors included in the final model.

### Statistical Analysis

Data were analyzed using SPSS version 17.0 (SPSS Inc., Chicago. USA). For all statistical testing, we used a 2-sided significance level (alpha) of 0.05. Survival curves were depicted using the Kaplan-Meier method and compared using the log-rank test. Multivariate analyses were based on Cox regression analysis.

We selected the optimum cutoff score for the number of lymph nodes examined using X-tile plots (version 3.6.1; Yale University School of Medicine, New Haven, CT, USA) 13.

For the development of nomogram, we randomly divided 70% of the whole data into a training cohort (n = 3198) and 30% into a validation cohort (n = 1368). A nomogram was formulated grounded on the results of multivariate analysis with the package of rms in R version 3.5.0.

All the factors included in the nomogram met the proportional-hazard assumption after reviewing the curves showing log[-log(S(t))]~t.

The median follow-up time was estimated by the method of reverse KM estimator.

The performance of the nomogram was assessed by concordance index (C-index) as well as by comparing nomogram-predicted versus observed Kaplan-Meier estimates of survival probability. Bootstraps with 1,000 resample were used for these Comparisons.The differences between the nomogram and the TNM stage systems were detected using the rcorrp.cens function in the R package Hmisc.

## Results

### Clinicopathologic Characteristics of Patients

A total of 4566 patients with non-metastatic esophageal carcinoma who had undergone surgery were included. Demographic and clinicopathologic characteristics of the study population were summarized in Table 1. ESCC and EAC accounted for 79.7% and 20.3% of the whole group,respectively. The 5-year survival rate was 40.5%, and the median follow-up time was 78.0 months (95%CI 75.9-80.1 months).

### Independent Prognostic Factors in the Training Cohort

A total of 3198 patients were assigned into the training cohort. Multivariate analyses demonstrated that age, gender, histology, grade, AJCC T stage, AJCC N stage, nodes examined, radiation and chemotherapy were independent risk factors for cancer-specific survival (CSS) (Table 2).

### Prognostic Nomogram for Cancer-specific Survival

**Figure 2:**
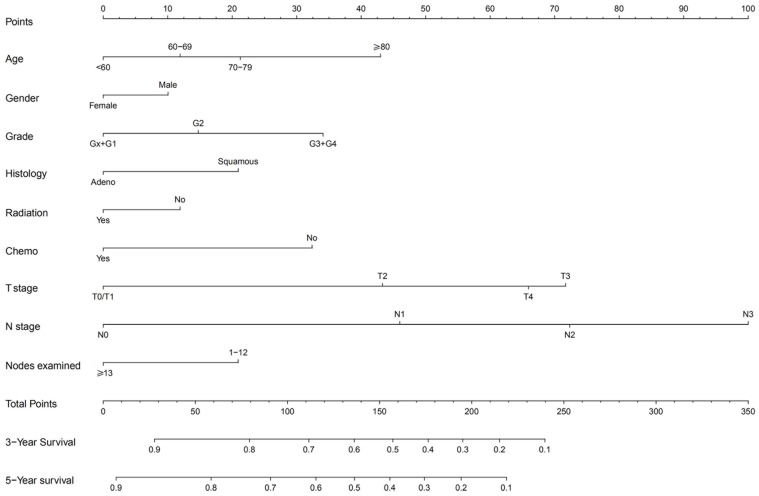
Non-metastatic esophageal carcinoma survival nomogram. (To use the nomogram, an individual patient^,^s value is located on each variable axis, and a line is drawn upward to determine the number of points received for each variable value. The sum of these numbers is located on the Total Points axis, and a line is drawn downward to the survival axes to determine the likelihood of 3- or 5-year survival).

The prognostic nomogram that integrated all significant independent factors for CSS in the training cohort was shown in Figure 2. The C-index for CSS prediction was 0.71 (95% CI, 0.70 to 0.72).

**Figure 3:**
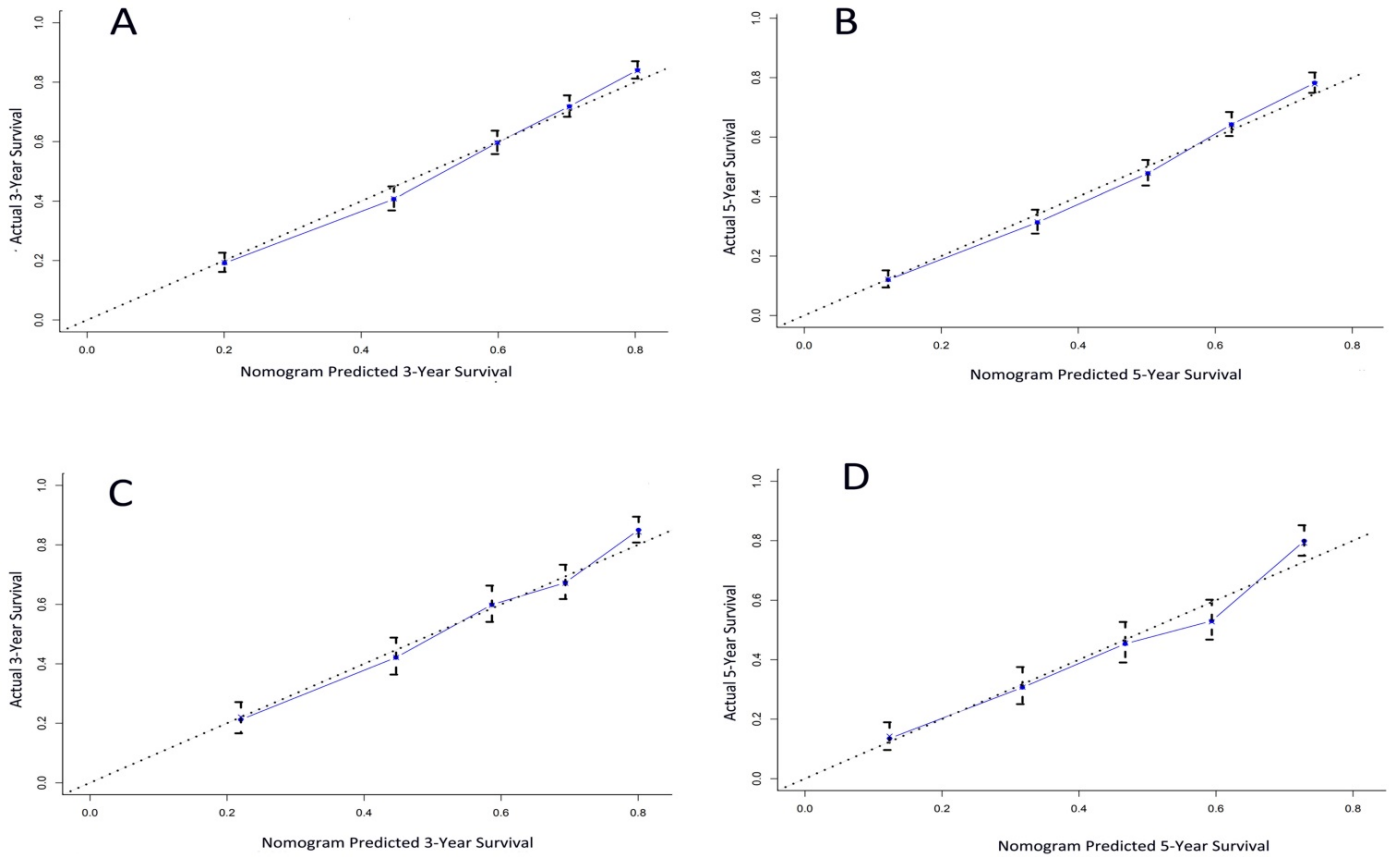
The calibration curve for predicting patient survival at (A) 3 years and (B) 5 years in the training cohort and at (C) 3 years and (D) 5 years in the validation cohort. Nomogram-predicted probability of overall survival is plotted on the x-axis; actual overall survival is plotted on the y-axis.

The calibration plot for the probability of survival at 3- and 5- year after surgery showed an optimal agreement between the prediction by nomogram and actual observation (Figure 3A and 3B).

### Comparison of Predictive Accuracy Between Nomogram and TNM Staging System

Our nomogram showed better accuracy in predicting 3-and 5-year survival in the training cohort. The C-index of the nomogram was 0.71, which was significantly higher (P<0.001) than the AJCC seventh edition staging system (0.67), the AJCC sixth edition staging system (0.64). The results suggested that the nomogram was a useful predictor for survival of patients with esophageal carcinoma in the training cohort.

### Validation of Predictive Accuracy of the Nomogram

A total of 1368 patients were assigned into the validation cohort. The C-index of the nomogram for predicting CSS was 0.70 (95% CI, 0.68 to 0.72), and a calibration curve showed good agreement between prediction and observation in the probability of 3- and 5-year survival (Figure 3C and 3D).

The C-index of nomogram was significantly higher (P< 0.001) than the AJCC seventh editing staging system (0.66), and the AJCC sixth edition staging system (0.65).

### Prognostic Nomogram for CSS in EAC and ESCC subgroups

In the EAC cohort, the prognostic nomogram that integrated all significant independent factors for CSS was displayed in supplementary figure 1. The C-index was 0.72 (95% CI, 0.71 to 0.73), which is significantly higher (P<0.001) than the AJCC seventh editing staging system (0.68), and the AJCC sixth edition staging system (0.66).

Similarly, the prognostic nomogram for CSS in the ESCC cohort was shown in supplementary figure 2. The C-index was 0.67 (95% CI, 0.65 to 0.70), which was significantly higher (P< 0.001) than the AJCC seventh edition staging system (0.62), and the AJCC sixth edition staging system (0.60).

The calibration curve for the probability of survival at 3 or 5 year suggested a satisfactory agreement between the prediction by nomogram and actual observation (Supplementary Figure 3A-D).

## Discussion

In this study, a prognostic nomogram based on large population database for patients with nMEC after surgery was constructed. The nomogram performed well in predicting 3-and 5-year cancer specific survival, which was supported by the C-index (0.71 and 0.70 for the training and validation cohorts, respectively) as well as the calibration plot. When compared with AJCC TNM staging systems, the nomogram demonstrated superior predictive accuracy for CSS.

Debate continued about the best strategies for the construction of nomogram in patients with esophageal cancer. In 2016, based on the SEER database, Cao et al. 8 constructed a nomogram for patients with esophageal cancer who underwent esophagectomy. The prognostic model included age, race, histology, tumor site, tumor size, grade, depth of invasion, number of positive nodes and the retrieved nodes. It exhibited a good survival prediction for those patients (C-index= 0.716). However, the main weakness of that study was the inadequate median follow-up time (28 months, range 3 to 276 months), which weakened its ability to predict long-term prognosis. Besides, the author made no attempt to evaluate performance of the nomogram in different histology subtypes. Moreover, the nomogram did not include information about radiation and systemic chemotherapy.

In contrast to the earlier finding, the median follow-up time in the present study was 78 months. Besides, we excluded race and tumor site from the nomogram due to their lack of statistical significance with survival. Lastly, considering the impact of radiation and chemotherapy on prognosis, we included these factors in order to provide more accuracy in survival prediction.

ESCC and EAC represented two primary histological subtypes of esophageal malignancy, with significant differences in epidemiology, tumor characteristics and genetic features 14, 15. Therefore, it is essential to assess the performance of the nomogram in the two subtypes separately. The C-index of nomogram in patients with EAC and ESCC was 0.72 and 0.67, respectively, which was both significantly higher than TNM staging system.

The conclusion of this study should be interpreted in caution because of the inherent limitation of SEER database such as the unrecorded variables, variations in data coding and reporting, and migration of patients in and out of SEER registry areas. Additionally, an entire sector of cancer management is unaccounted for in this database, as pharmaceutical information like chemotherapy. Besides, some confounding bias and selection bias also should be taken into account when interpreting the results from this observational study based on SEER 16.

## Conclusion

We developed and validated a prognostic nomogram to provide an individual survival prediction for nMEC patients who underwent surgery. Compared with the TNM staging system, our nomogram exhibits a better prognostic discrimination and survival prediction. It is likely that these results would play a supplementary role to the current staging system and help to identify the high risk population after surgery.

## Supplementary Material

Supplementary figures.

## Figures and Tables

**Table 1:** Demographics and Clinicopathologic Characteristics of Patients With non-metastatic esophageal carcinoma

		All cases			Training			Validation	
Variables		N	%		N	%		N	%
**Age**	**<60**	1709	37.4		1200	37.5		509	37.2
	**60-69**	1754	38.4		1221	38.2		533	39.0
	**70-79**	931	20.4		659	20.6		272	19.9
	**≥80**	172	3.8		118	3.7		54	3.9
**Race**	**White**	4129	90.4		2892	90.4		1237	90.4
	**Black**	246	5.4		170	5.3		76	5.6
	**Others**	186	4.1		131	4.1		55	4.0
	**Unknown**	5	0.1		5	0.2		0	0.0
**Gender**	**Male**	3881	85.0		2736	85.6		1145	83.7
	**Female**	685	15.0		462	14.4		223	16.3
**Location**	**Proximal**	86	1.9		65	2.0		21	1.5
	**Middle**	657	14.4		455	14.2		202	14.8
	**Distal**	3541	77.6		2484	77.7		1057	77.3
	**Unknown**	282	6.2		194	6.1		88	6.4
**Histology**	**Adeno**	3637	79.7		2553	79.8		1084	79.2
	**Squamous**	929	20.3		645	20.2		284	20.8
**Grade**	**GX/1**	772	16.9		556	17.4		216	15.8
	**G2**	1804	39.5		1260	39.4		544	39.8
	**G3/4**	1990	43.6		1382	43.2		608	44.4
**T stage**	**T0/1**	1327	29.1		938	29.3		389	28.4
	**T2**	729	16.0		521	16.3		208	15.2
	**T3**	2256	49.4		1560	48.8		696	50.9
	**T4**	254	5.6		199	6.2		75	5.5
**N stage**	**N0**	2871	62.9		2024	63.3		847	61.9
	**N1**	981	21.5		684	21.4		297	21.7
	**N2**	484	10.6		343	10.7		141	10.3
	**N3**	230	5.0		147	4.6		83	6.1
**Radiation**	**Yes**	2748	60.2		1936	60.5		812	59.4
	**No**	1818	39.8		1262	39.5		556	40.6
**Chemo**	**Yes**	2989	65.5		2104	65.8		885	64.7
	**No**	1577	34.5		1094	34.2		483	35.3
**Nodes examined**	**1-12**	2328	51.0		1612	50.4		716	52.3
	**≥13**	2238	49.0		1586	49.6		652	47.7
**Total**		**4566**			**3198**			**1368**	

**Table 2:** Multivariate Cox Analysis of the training cohort

Variable		Cancer-specific survival			
		HR	95% CI		P value
**Age**					<0.001
	**<60**	Ref			
	**60-69**	1.18	1.06	1.32	
	**70-79**	1.35	1.18	1.55	
	**≥80**	1.85	1.44	2.38	
**Race**					0.306
	**White**	Ref			
	**Black**	1.02	0.82	1.28	
	**Others**	0.95	0.74	1.23	
	**Unknown**	0.39	0.06	2.78	
**Gender**					0.045
	**Male**	Ref			
	**Female**	0.85	0.73	0.99	
**Histology**					<0.001
	**Adeno**	Ref			
	**Squamous**	1.18	1.01	1.37	
**Grade**					<0.001
	**GX/1**	Ref			
	**G2**	1.233	1.05	1.45	
	**G3/4**	1.63	1.39	1.91	
**Location**					0.310
	**Proximal**	Ref			
	**Mid**	1.09	0.74	1.59	
	**Distal**	0.80	0.55	1.16	
	**Unknown**	1.15	0.76	1.73	
**T stage**					<0.001
	**T0/1**	Ref			
	**T2**	1.90	1.58	2.28	
	**T3**	2.90	2.47	3.40	
	**T4**	2.59	2.03	3.3	
**N stage**					<0.001
	**N0**	Ref			
	**N1**	1.95	1.73	2.20	
	**N2**	2.85	2.46	3.30	
	**N3**	4.17	3.43	5.08	
**Nodes examined**					<0.001
	**1-12**	Ref			
	**≥13**	0.74	0.67	0.82	
**Radiation**					0.024
	**Yes**	Ref			
	**No**	1.18	1	1.40	
**Chemo**					<0.001
	**No**	Ref			
	**Yes**	0.63	0.53	0.76	

## Abbreviations

ESCC: Esophageal squamous carcinoma
EAC: Esophageal adenocarcinoma
SEER: Surveillance, Epidemiology and End Results
OS: Overall survival
CSS: Cancer specific survival
HR: Hazard ratio
CI: Confidence interval
nMEC: non-metastatic esophageal carcinoma
