# Supplementary material for: Development and Validation of an Individualized Nomogram for Predicting Survival in Patients with Esophageal Carcinoma after Resection
Source: J Cancer. 2020 Apr 6;11(14):4023–9. doi: 10.7150/jca.40767 (PMC7196250; doi:10.7150/jca.40767)
Supplement: Supplementary file 1 — Supplementary figures. [file jcav11p4023s1.pdf]

Supplementary File

Figure.1 Non-metastatic esophageal adenocarcinoma survival nomogram

Figure.2 Non-metastatic esophageal squamous cell carcinoma survival nomogram

Figure.3 The calibration curve for predicting patient survival at (A) 3 years and (B) 5 years in the EAC cohort and at (C) 3 years and (D) 5 years in the ESCC cohort. Nomogram-predicted probability of overall survival is plotted on the x-axis; actual overall survival is plotted on the y-axis.

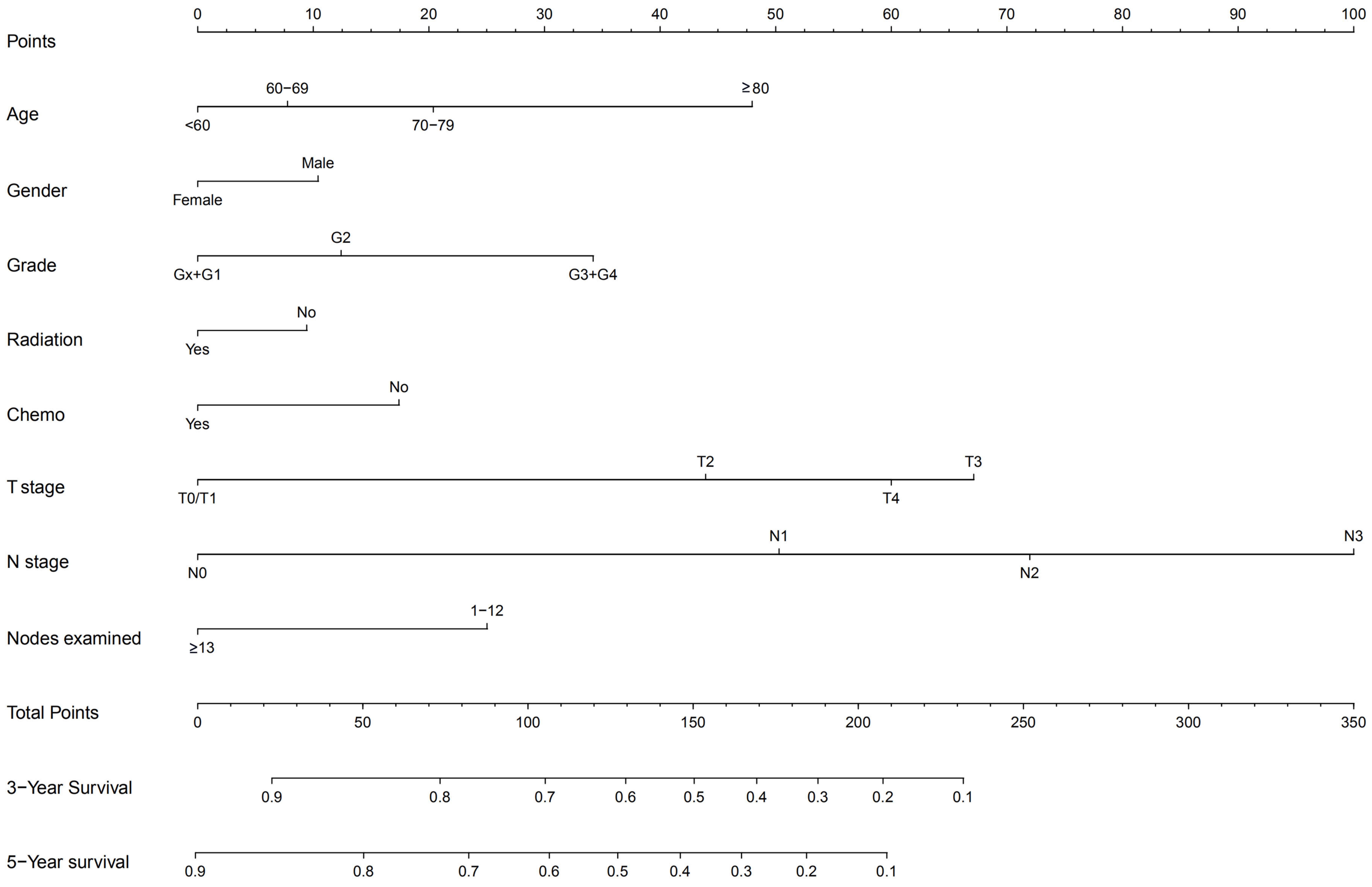
Points
0
10
20
30
40
50
60
70
80
90
100
Age
60−69
≥80
<60
70−79
Gender
Male
Female
Grade
G2
Gx+G1
G3+G4
Radiation
No
Yes
Chemo
No
Yes
T stage
T2
T3
T0/T1
T4
N stage
N1
N3
N0
N2
Nodes examined
1−12
≥13
Total Points
0
50
100
150
200
250
300
350
3−Year Survival
0.9
0.8
0.7
0.6
0.5
0.4
0.3
0.2
0.1
5−Year survival
0.9
0.8
0.7
0.6
0.5
0.4
0.3
0.2
0.1

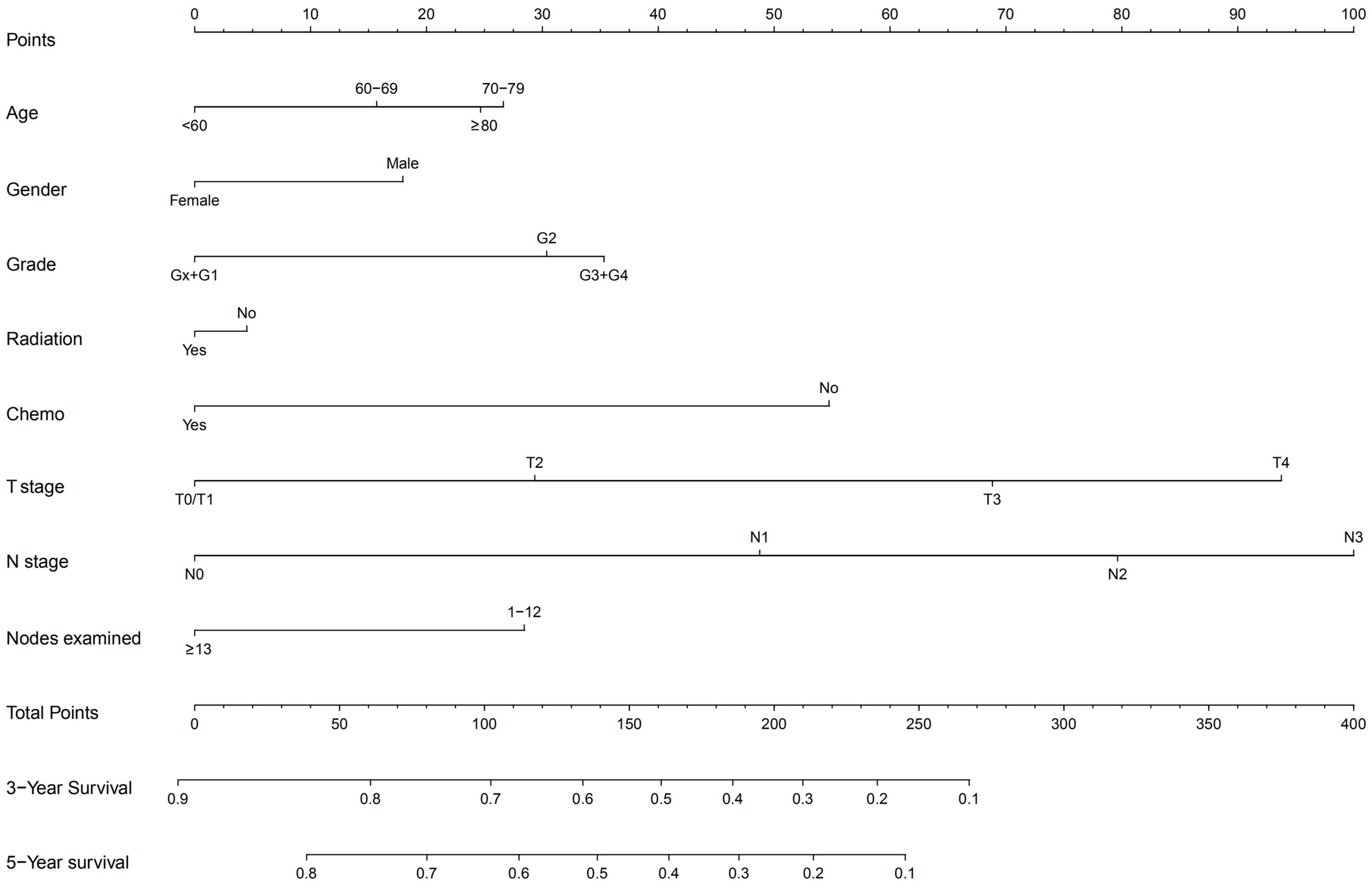
Points
0
10
20
30
40
50
60
70
80
90
100
Age
60−69
70−79
<60
≥80
Gender
Male
Female
Grade
G2
Gx+G1
G3+G4
Radiation
No
Yes
Chemo
No
Yes
T stage
T2
T4
T0/T1
T3
N stage
N1
N3
N0
N2
Nodes examined
1−12
≥13
Total Points
0
50
100
150
200
250
300
350
400
3−Year Survival
0.9
0.8
0.7
0.6
0.5
0.4
0.3
0.2
0.1
5−Year survival
0.8
0.7
0.6
0.5
0.4
0.3
0.2
0.1

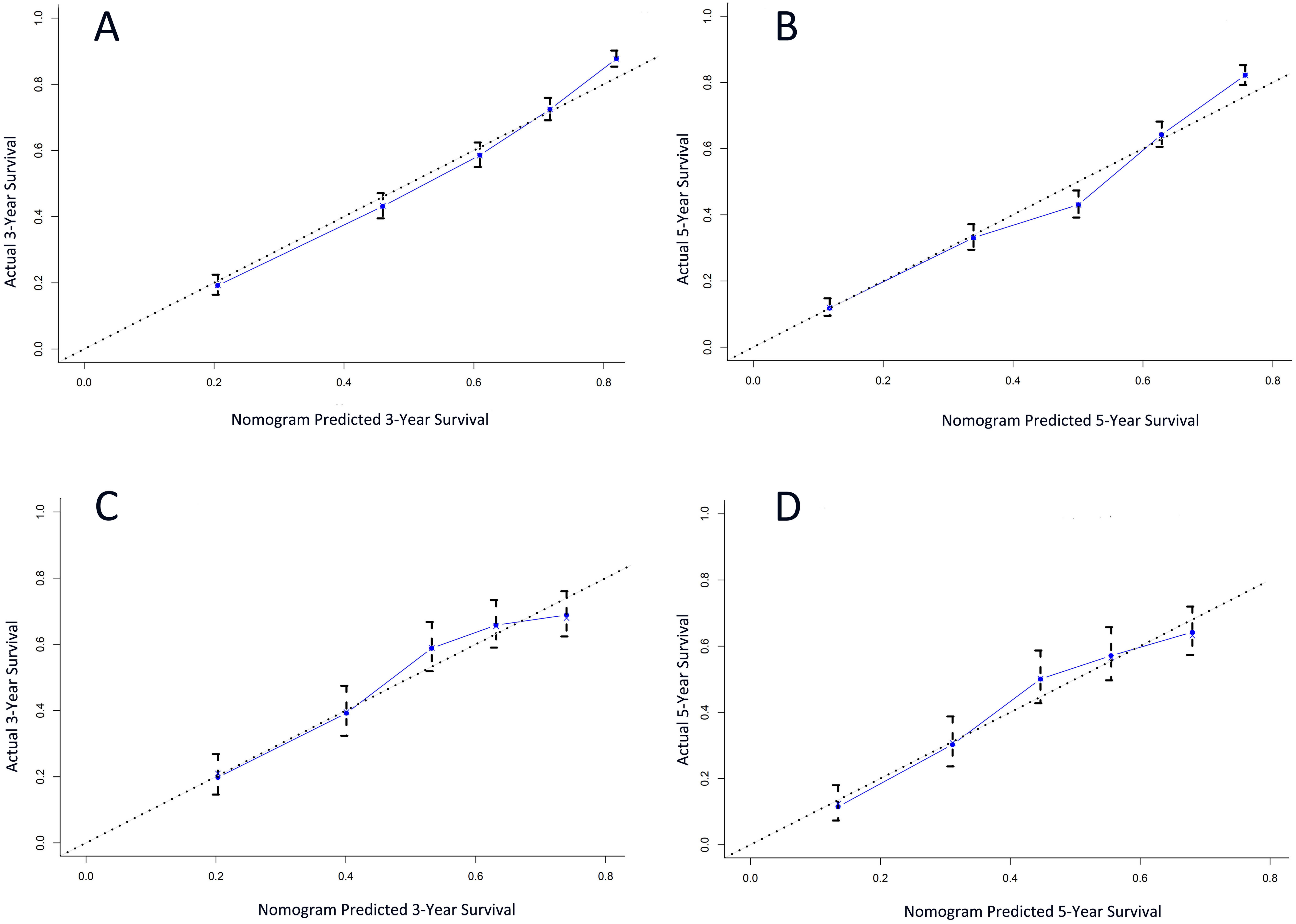
A
B
C
D
Actual 3-Year Survival
Actual 5-Year Survival
Nomogram Predicted 3-Year Survival
Nomogram Predicted 5-Year Survival
0.0
0.2
0.4
0.6
0.8
1.0
